# Three Cases of Trichnasis in Clay County, Ill.

**Published:** 1869-05-15

**Authors:** E. W. Boyles

**Affiliations:** Clay City, Ill.


### *Three Cases of Trichnasis in Clay Co., Illinois.*

BY E. W. BOYLES, M. D., OF CLAY CITY, ILL.

A family by the name of Stafford, consisting of the mother and three daughters, moved into this county about the first of March last. They had formerly lived in Edgwood, in the adjoining county of Effingham. Shortly after coming into this county the three daughters, aged respectively six, twelve and sixteen years, were taken sick; they first complained of weakness, soreness and tenderness of muscles, stiffness of the limbs, which increased till the two oldest were soon prostrated. There was, also, considerable gastric derangement present, and fever of a low form with decided exacerbations, in the evening ensued.

Dr. Anderson, of this county, was called in, and diagnosed the disease *trichinasis*, (i e) he said if it was not that he did not know what it was. Dr. Duncan was called in consultation, and subsequently Drs. Green and McKee, of Louisville. They all arrived at the same conclusion, that the disease was *trichinasis*.

In about a fortnight from the time of attack, the girl aged twelve years died; a postmortem was held, at which the Doctors mentioned above, and Dr. J. W. Hitchcock, of Louisville, in this county, officiated, but having no glass of any considerable power, no satisfactory conclusions were arrived at. Dr. Hitchcock removed a portion of the biceps muscle of the left arm, and sent it to me for microscopical examination. Upon placing a small portion under the glass the parasites *trichina spiralis* were to be seen in great numbers. I tried several specimens and never failed to find them in great abundance; they were of various sizes, but none encysted. I then enclosed a small portion of the muscle in a letter, and sent it to Dr. J. Adams Allen, of Chicago, requesting him to examine it with the microscope and report to me the results. I did not state to him that I had made any examination of it myself. In a few days I received a letter from Walter Hay, M. D., of Chicago, from which I make the following extracts: "Your letter of the 13th inst. was handed to me yesterday by Dr. Allen, with the request that I would examine the specimen enclosed and favor him with my opinion of its condition. * * * I can assure you that I have never seen any specimen which even approximated to this in the number of parasites. * * * After measuring a cubic tenth of an inch (.1) and dividing this again into eight (8) portions, I counted in one of these eights forty-five trichina, all free, none encysted."

The circumstances connected with the cases, as I learned then of Drs. Anderson, Hitchcock and McKee, are these: The mother is an invalid, and previous to their moving into this county they bought pork of a grocer in Edgewood, and the elder sister states that frequently when she went to cook the meat (cured ham,) she would slice off small bits of it and eat it raw, and that she gave some to the younger sister, and they ate of it in the same way; the sister that died being particularly fond of it ate more than she or the younger sister; the younger sister ate but little, and she was only slightly affected with the disease, the mother never ate any of the meat raw and never had the disease.

The oldest sister is still living, but is in a very precarious condition. It is stated that the grocer bought his pork in Chicago.

